# RETRACTED: Ikram et al. Natural Dietary Supplementation of Curcumin Protects Mice Brains Against Ethanol-Induced Oxidative Stress-Mediated Neurodegeneration and Memory Impairment via Nrf2/TLR4/RAGE Signaling. *Nutrients* 2019, *11*, 1082

**DOI:** 10.3390/nu18030416

**Published:** 2026-01-27

**Authors:** Muhammad Ikram, Kamran Saeed, Amjad Khan, Tahir Muhammad, Muhammad Sohail Khan, Min Gi Jo, Shafiq Ur Rehman, Myeong Ok Kim

**Affiliations:** Division of Applied Life Science (BK 21), College of Natural Sciences, Gyeongsang National University, Jinju 52828, Republic of Korea; qazafi417@gnu.ac.kr (M.I.); kamran.biochem@gnu.ac.kr (K.S.); amjadkhan@gnu.ac.kr (A.K.); mtahir.khan@gnu.ac.kr (T.M.); sohail.bannu@gnu.ac.kr (M.S.K.); mingi.cho@gnu.ac.kr (M.G.J.); shafiq12@gnu.ac.kr (S.U.R.)

The journal retracts the article “Natural Dietary Supplementation of Curcumin Protects Mice Brains against Ethanol-Induced Oxidative Stress-Mediated Neurodegeneration and Memory Impairment via Nrf2/TLR4/RAGE Signaling” [1] cited above.

Following publication, concerns were brought to the attention of the Editorial Office regarding the integrity of a number of images presented in this publication [1].

Adhering to our standard procedure, an investigation was conducted by the Editorial Office and Editorial Board that identified indications of inappropriate editing and partial duplication within Figures 1F, 2A,C,D, 3A,C,D, 4D, 5B and 6A,B presented in this article. While the authors collaborated within this process, full raw material meeting the journals requirements for original images could not be provided for Editorial Board evaluation (https://www.mdpi.com/journal/nutrients/instructions#oriimages (accessed on 18 November 2025)). Consequently, the Editorial Board has lost confidence in the reliability of the findings and has decided to retract this publication, as per MDPI’s retraction policy (https://www.mdpi.com/ethics#_bookmark30 (accessed on 18 November 2025)).

This retraction was approved by the Editor-in-Chief of the *Nutrients* journal.

The authors did not agree to this retraction.
